# Development of an HPLC method using relative molar sensitivity for the measurement of blood concentrations of nine pharmaceutical compounds

**DOI:** 10.1186/s40780-024-00358-6

**Published:** 2024-07-05

**Authors:** Takashi Ohtsuki, Yi Huang, Ayane Kamiya, Yuki Nakayama, Miyuki Matsushita, Satoru Morikawa, Hiroshi Matsufuji

**Affiliations:** 1 Department of Food Science and Technology, College of Bioresource Sciences, Nihon University, Kameino, Fujisawa, 1866 Kanagawa Japan; 2 Hitachi High-Tech Science Corporation, 1-17-1, Toranomon, Minato-ku, Tokyo, Japan

**Keywords:** Relative molar sensitivity, HPLC, Carbamazepine, Phenytoin, Voriconazole, Lamotrigine, Meropenem, Mycophenolic acid, Linezolid, Vancomycin, Caffeine, Therapeutic drug monitoring

## Abstract

We developed a reliable high-performance liquid chromatographic analysis method using a relative molar sensitivity (RMS) technique that does not require an authentic, identical reference analyte material to quantify blood serum carbamazepine, phenytoin, voriconazole, lamotrigine, meropenem, mycophenolic acid, linezolid, vancomycin, and caffeine levels for routine blood concentration measurements. Carbamazepine and caffeine were also used as non-analyte reference materials to calculate the RMS of each analyte. The RMS was calculated from the ratio of the slope of the calibration equation (analyte/non-analyte reference material), then used to quantify analytes in control serum samples spiked with carbamazepine, phenytoin, voriconazole, meropenem, mycophenolic acid, linezolid or vancomycin. In addition, the concentrations of these six drugs in control serum samples determined by the proposed RMS method agreed well with that obtained using a conventional method. The proposed RMS method is a promising tool for the clinical determination of nine drugs, given the accuracy, precision, and efficiency of quantifying these analytes.

## Background

The efficacy of and adverse reactions caused by typical drugs used in drug therapy may differ between individuals. Some drugs administered at the same dosage can cause different effects resulting from differences in blood drug concentrations due to the weight, age, pre-existing medical conditions, concomitant medications, and other factors unique to each patient. Drugs with narrow effective and safe concentration ranges in particular require set dosage regimens to maintain their range of effective blood concentrations and this control can be achieved using therapeutic drug monitoring (TDM). In TDM, blood drug concentrations are measured to provide an effective and safe drug therapy for individual patients, such as determining the therapeutic and side effects of administered drugs. This information is crucial for optimizing the dosage and the administration method for each patient. Blood concentrations are mainly measured by immunological assays such as enzyme-linked immunosorbent assay (ELISA) [1, 2], fluorescence polarization immunoassay (FPIA) [3], and enzyme multiplied immunoassay technique (EMIT) [4]. However, when using an immunoassay kit, if the antibody for the analyte is not commercially available, the measurement cannot be performed in medical institutions. In addition, immunoassay kits can process many specimens simultaneously but if there is only a small number of specimens, the kit cannot be used efficiently. The measurement of drug concentrations in TDM requires facile, fast and accurate measurement of the blood concentrations of therapeutic drugs to quickly determine the blood therapeutic drug concentration and to optimize the dosage and administration.

High-performance liquid chromatography (HPLC) is a fast, straightforward technique that offers outstanding recovery and high precision for various pharmaceutical compounds, ensuring accuracy and precision [5]. In addition, optimized HPLC conditions allow the separation of pharmaceuticals, chemicals, and metabolites in biological specimens. Several HPLC methods have been developed to determine TDM-related medicines in human specimens [6–11].

HPLC systems requiring little operational expertise have recently been developed for routine use in medical institutions [12, 13]. HPLC is thus gaining importance in the measurement of drug concentrations as it allows the real-time evaluation of therapeutic drug concentrations in blood.

Quantification using HPLC generally requires an absolute calibration curve method. The reliability of the analytical value obtained using this method requires a quantitative reference material that is identical to the analyte and whose purity is known accurately. However, the variety of analytes that must be measured clinically makes it difficult to obtain quantitative reference materials, and even when they are available, they often deviate significantly from the indicated purity due to stability issues and moisture absorption. These factors present a significant challenge for precise quantification using HPLC. Reference materials such as certified reference materials (CRMs) with a defined exact purity are necessary for accurate quantification but are not available for all analytes. The availability of quantitative reference materials remains problematic.

We are addressing these issues related to quantitative reference materials by establishing a quantitative HPLC analysis method using relative molar sensitivity (RMS). RMS is a coefficient defined as the response ratio of an analyte to that of a CRM of a non-analyte reference material different from the analyte, per unit mole. In this RMS-based quantification method, if the exact RMS between the analyte and a CRM of the non-analyte is known, one can accurately quantify various analytes using the CRM of the non-analyte based on the relationship between the response values and the RMSs of the CRM of the non-analyte and of the analyte. In addition, because the RMS is calculated based on the absolute purity of the analyte and the CRM of the non-analyte with traceability to the international system of units (SI), the reliability of the obtained quantitative value is high. Therefore, this method holds promise for solving the abovementioned problems related to analyte reference materials, and for increasing the speed and simplifying the quantitative analysis at reduced cost. This method has been used to quantify food compounds [14–18], natural products [19–23], polycyclic aromatic hydrocarbons [24], major food additives [25–29], and the TDM of drugs such as carbamazepine and phenytoin [30].

**Fig. 1:**
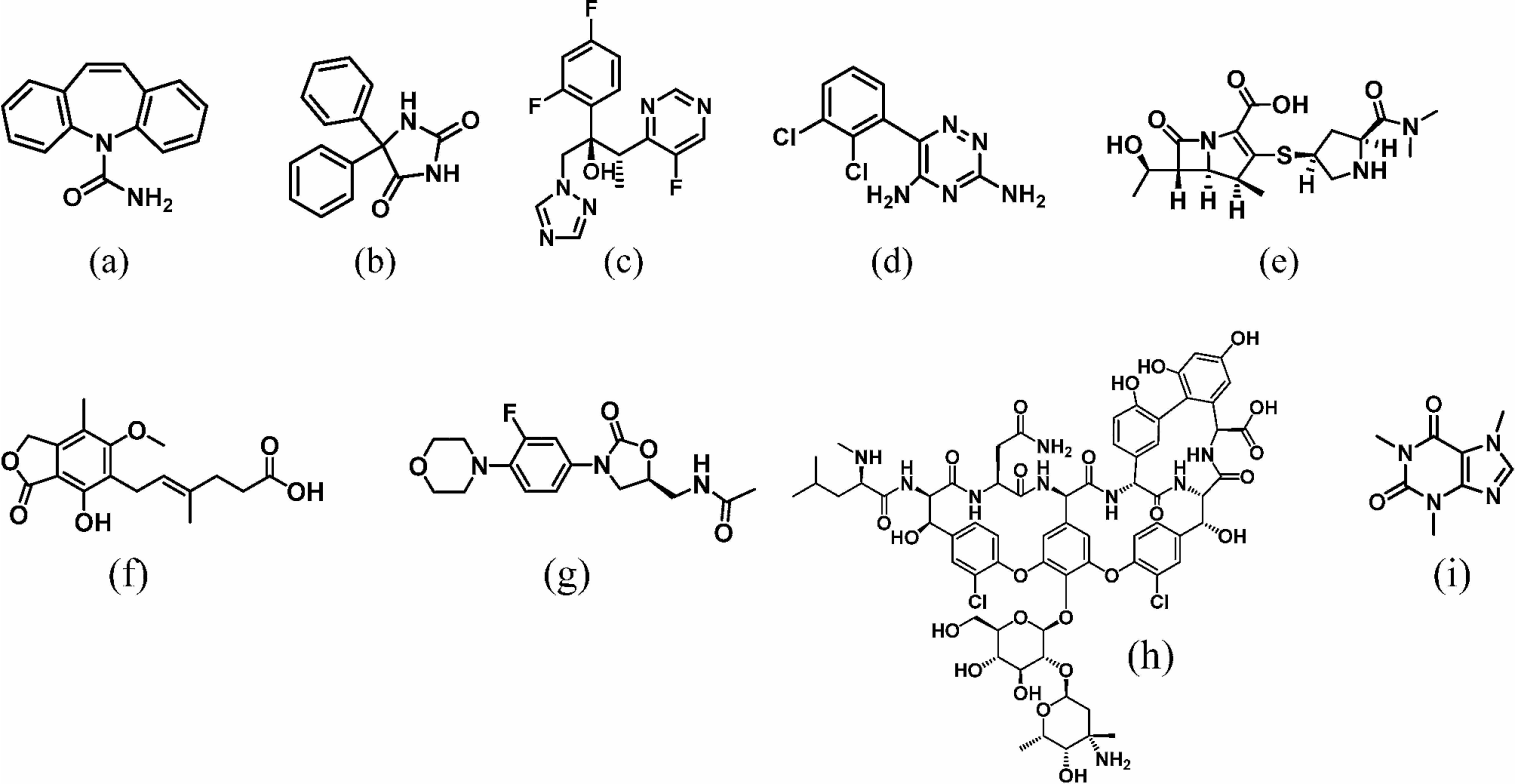
Chemical structures of the nine analytes (**a**) carbamazepine, (**b**) phenytoin, (**c**) voriconazole, (**d**) lamotrigine, (**e**) meropenem, (**f**) mycophenolic acid, (**g**) linezolid, (**h**) vancomycin, (**i**) caffeine

In this study, we developed an HPLC method using RMS for quantifying carbamazepine, phenytoin voriconazole, lamotrigine, meropenem, mycophenolic acid, linezolid, vancomycin and caffeine (Fig. 1) to improve the reliability and efficiency of drug blood concentration measurements. In addition, using control serum samples spiked with each analyte, the RMS method was compared with a conventional HPLC method using analyte reference materials.

## Methods

### Samples and reagents

The CRM solutions of voriconazole (concentration: 5 μg/mL), carbamazepine, phenytoin, lamotrigine, meropenem, and mycophenolic acid (20 μg/mL each), linezolid (50 μg/mL), and vancomycin and caffeine (100 μg/mL each) were obtained from Hitachi High-Tech Science Co., Ltd. (Tokyo, Japan). The control serum samples spiked with each analyte used liquid control serum I (Fujifilm Wako Pure Chemical Industries, Ltd., Osaka, Japan) and were prepared by Hitachi High-Tech Science Co., Ltd. Other reagents and solvents were of special grade or HPLC grade.

### Instruments

An AUW220D semimicro balance (Shimadzu Corporation, Kyoto, Japan) was used to prepare the calibration standard solutions of each analyte for HPLC analysis. Analytical HPLC was performed using the evaluation HPLC system for LM1010 (Hitachi High-Tech Science Co., Ltd.).

A model 3740 centrifuge (Kubota Corporation, Tokyo, Japan) was used for the pre-treatment of analyte-spiked liquid control serum I samples with a solid-phase extraction method using spin columns to provide HPLC test solutions.

### Preparation of calibration standard solutions of the analytes and non-analyte reference materials

Calibration standard solutions of carbamazepine, lamotrigine, phenytoin, voriconazole, and mycophenolic acid were prepared by accurately diluting their respective calibration standard solutions with 50% (w/w) acetonitrile to 6–10 concentrations. For linezolid and vancomycin, the calibration standard solutions were prepared using 30% (w/w) acetonitrile. For caffeine, the calibration standard solutions were prepared using 10% (w/w) acetonitrile. Three sets of calibration standard solutions were prepared for each target compound.

### HPLC analysis of the calibration standard solutions

The calibration standard solutions of each analyte were analyzed using the evaluation HPLC system for LM1010 with monitoring at 220 nm (phenytoin, lamotrigine, mycophenolic acid), 235 nm (vancomycin), 250 nm (linezolid), 254 nm (voriconazole), 270 nm (caffeine), 280 nm (carbamazepine), and 295 nm (meropenem) using two dedicated mobile phases, Mobile Phase A and Mobile Phase B (Hitachi High-Tech Science Co., Ltd.) and a LaChrome LM TypeA analytical column (Hitachi High-Tech Science Co., Ltd.) according to the HPLC methods for each analyte of LM1010 and literatures [12, 13, 31]. The measurement of each sample was complete after 7 min, including flushing and re-equilibration of the analytical column.

### Calculation of RMSs of the analytes relative to the non-analyte reference material

The RMS of each analyte relative to the non-analyte reference material based on the plotted HPLC results was calculated from the ratio of the slope of the calibration equation (analyte/alternative reference material) shown in Eq. (1) after the calibration curve passed through the origin for each analyte and the non-analyte reference material [15]. Carbamazepine and caffeine were used as non-analyte reference materials.

1 $${\rm{RMS}} = \frac{{{\rm{Slope}}\,{\rm{of}}\,{\rm{calibration}}\,{\rm{equation}}\,{\rm{for}}\,{\rm{analyte}}}}{{{\rm{Slope}}\,{\rm{of}}\,{\rm{calibration}}\,{\rm{equation}}\,{\rm{for}}\,{\rm{alternative}}\,{\rm{reference}}\,{\rm{material}}}}$$

### Analysis of control serum samples spiked with each analyte using the RMS method

Control serum samples spiked with each analyte (carbamazepine, phenytoin, voriconazole, meropenem, mycophenolic acid, linezolid or vancomycin) at one or two concentrations was pretreated with reference to the literature [12, 13]. Briefly, 150 μL of control serum samples filtered through a 0.45 μm syringe filter was treated using the solid-phase extraction method with a spin column optimized for each analyte, and the obtained solutions were used as test solutions. Three sets of test solutions were used for each drug and concentration. In the RMS method, the concentration of each analyte in the test solution was determined from the peak area obtained for each analyte using a calibration curve of the non-analyte reference material (carbamazepine or caffeine) and the RMS value. For the conventional method, the calibration curve for each analyte was used to determine concentration. The concentration of each analyte in the control serum sample was calculated using Eq. (2):

2 $${\rm{Concentration}}\left( {\mu {\rm{g/mL}}} \right){\rm{ = C }} \times \frac{{\rm{1}}}{{{\rm{ RMS}}}} \times \frac{{{{\rm{M}}_{\rm{A}}}}}{{{\rm{1000}}}}$$

where C is the concentration of each analyte in the test solution determined from the calibration curve of the non-analyte reference material (μmol/L), M_A_ is the molecular weight of the analyte (g/mol), and RMS is the ratio of the RMS of the analyte to that of the non-analyte reference material.

In the conventional method, each analyte in the sample was quantified using the same test solution. The concentrations of analytes in the test solutions (μmol/L) were determined by integrating the peak areas of each analyte with the calibration curve for the authentic reference material. The concentration of the analyte in each sample was calculated using Eq. (3):

3 $${\rm{ Concentration}}\left( {\mu {\rm{g/mL}}} \right){\rm{ = C}} \times \frac{{{{\rm{M}}_{\rm{A}}}}}{{{\rm{1000}}}}$$

## Results and discussion

### Linearity

**Fig. 2:**
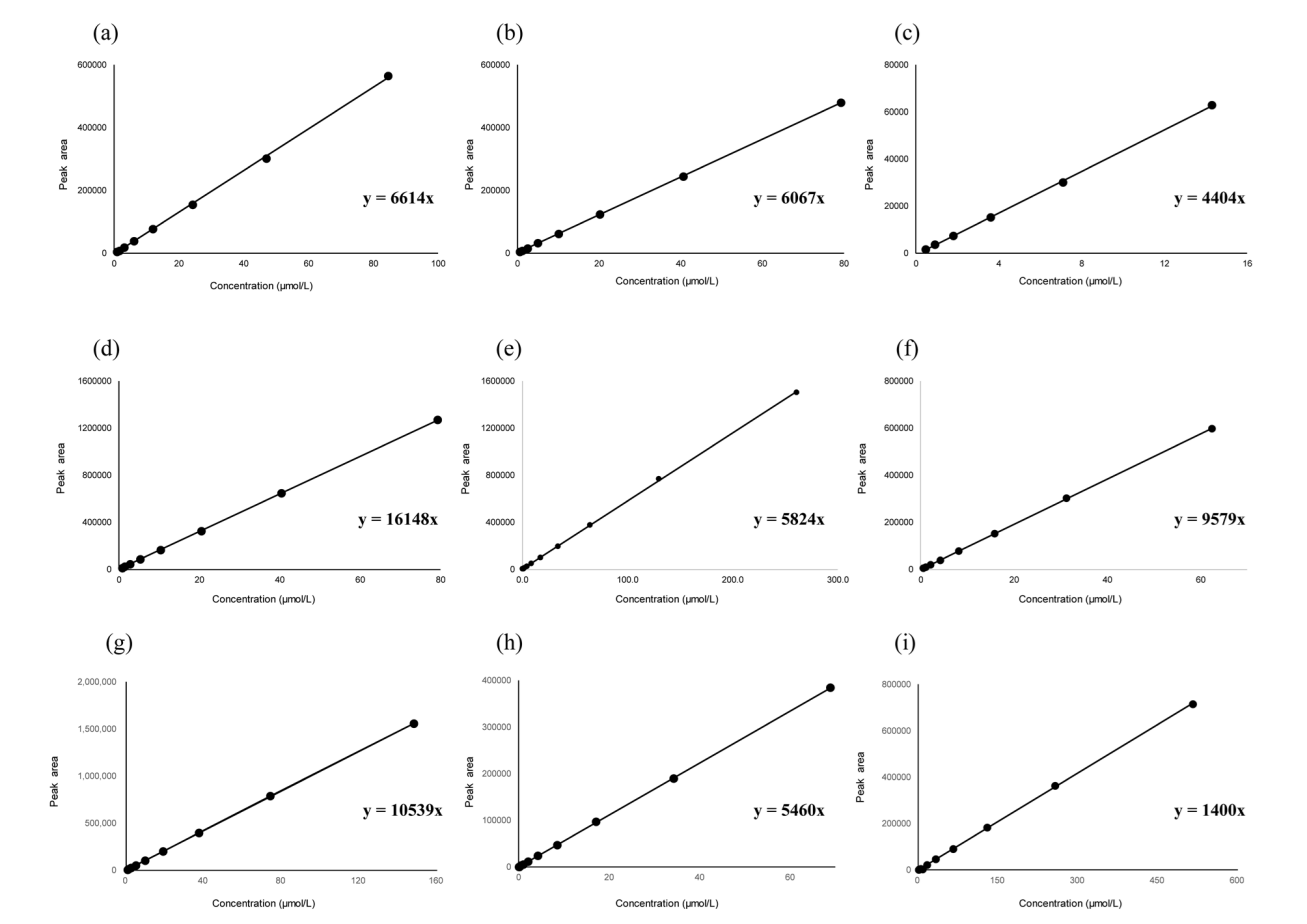
Typical calibration curves for each analyte. (**a**) carbamazepine, (**b**) phenytoin, (**c**) voriconazole, (**d**) lamotrigine, (**e**) meropenem, (**f**) mycophenolic acid, (**g**) linezolid, (**h**) vancomycin, (**i**) caffeine The concentrations of the calibration standard solutions for each analyte are as follows: carbamazepine: 0.9, 1.8, 3.4, 6.4, 12, 24, 45, and 85 μmol/L; phenytoin: 0.7, 1.4, 2.6, 4.9, 9.7, 19, 39, and 79 μmol/L; voriconazole: 0.15, 0.29, 0.63, 1.3, 2.5, and 5.0 μmol/L; lamotrigine: 0.7, 1.3, 2.7, 5.2, 10, 20, 40, and 79 μmol/L; meropenem: 0.5, 1.0, 2.2, 4.3, 8.5, 17, 33, 66, 132, and 261 μmol/L; mycophenolic acid: 0.6, 1.2, 2.3, 4.4, 8.6, 17, 32, and 62 μmol/L; linezolid: 0.6, 1.1, 2.3, 4.6, 9.0, 18, 37, 74, and 148 μmol/L; vancomycin: 0.1, 0.3, 0.5, 1.1, 2.2, 4.3, 8.6, 17, 34, and 69 μmol/L; caffeine: 1.0, 1.9, 3.9, 7.9, 16, 32, 64, 129, 256, and 515 μmol/L

**Fig. 3:**
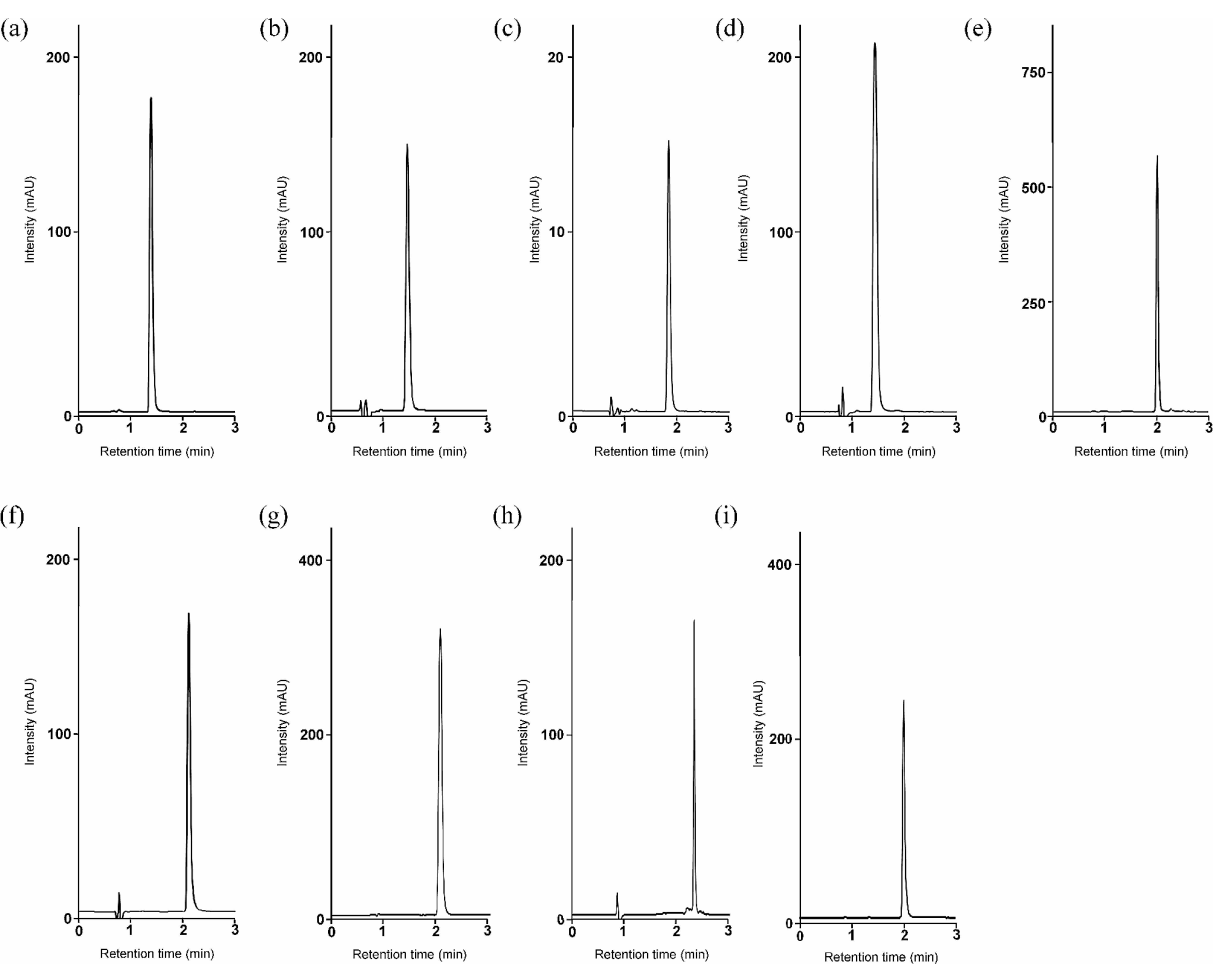
Typical chromatograms for each analyte (**a**) carbamazepine, (**b**) phenytoin, (**c**) voriconazole, (**d**) lamotrigine, (**e**) meropenem, (**f**) mycophenolic acid, (**g**) linezolid, (**h**) vancomycin, (**i**) caffeine

It is important to verify the linearity of the method because the concentration of the analyte should lie within the linear range of the calibration plot. First, we analyzed eight calibration standard solutions for carbamazepine, phenytoin, and lamotrigine, six concentrations for voriconazole, and 10 concentrations for meropenem, linezolid, vancomycin and caffeine, 9 concentrations for linezolid to determine the linearity and measurement range of the analytes and of the non-analyte reference materials. Accurate calibration curves were constructed based on the exact concentration of each calibration standard solution and the corresponding chromatographic peak areas. As shown in Table 1; Figs. 2 and 3, good linearity with a correlation coefficient (R^2^) > 0.999 was obtained for all calibration curves over the concentration ranges examined. In addition, the slopes of the calibration curves obtained in triplicate for the individual analyte and non-analyte reference materials showed no significant variations, with the relative standard deviation (RSD) ranging from 0.2 to 1.6%. The slope of the calibration equation is indicated by the mean value of the three obtained calibration equations for each analyte. The RSD was calculated from the slopes of the three calibration equations obtained for each analyte. In addition, the limits of quantification for carbamazepine, phenytoin, voriconazole, lamotrigine, meropenem, mycophenolic acid, linezolid, vancomycin, and caffeine were determined to be 0.9, 0.7, 0.15, 0.7, 0.5, 0.6, 0.6, 0.1, and 1.0 μmol/L, respectively, based on visual evaluation and the signal-to-noise ratio.

**Table 1:** Regression data for each analyte

	Calibration equation	Relative standard deviation (%)	Coefficient of determination	Concentration range of the calibration standard (μmol/L)
Carbamazepine	y = 6614 x	0.2	1.00	0.9–84.6
Phenytoin	y = 6067 x	1.0	1.00	0.7–79.3
Voriconazole	y = 4404 x	1.3	1.00	0.6–79.3
Lamotrigine	y = 16,148 x	1.1	1.00	0.7–79.3
Meropenem	y = 5824 x	0.5	1.00	0.5–261.0
Mycophenolic acid	y = 9579 x	0.2	1.00	0.6–62.4
Linezolid	y = 10,539 x	0.2	1.00	0.6-148.2
Vancomycin	y = 5460 x	1.6	1.00	0.1–69.0
Caffeine	y = 1400 x	0.6	0.999-1.00	1.0-515.0

### Calculation of RMS of six drugs against the non-analyte reference material

Calculating the RMS using HPLC coupled to ultraviolet-visible (UV-Vis) detector, requires determining the ratio of the slope of the calibration curve of the analyte to that of the non-analyte reference material. Carbamazepine and caffeine were used as non-analyte reference materials. As shown in Table 1, the slope of each analyte indicates that the mean slope values of the calibration curves for each analyte and the non-analyte reference material are appropriate for calculating RMS values. Therefore, the RMS of each analyte to the non-analyte reference material was calculated from the slope ratio (analyte/each non-analyte reference material) according to Eq. (1), and the results are shown in Table 2.

**Table 2:** RMS of each analyte to the two non-analyte reference materials

	Non-analyte reference material	
	Carbamazepine	Caffeine
Carbamazepine	1.00	4.72
Phenytoin	0.917	4.33
Voriconazole	0.666	3.15
Lamotrigine	2.44	11.5
Meropenem	0.881	4.16
Mycophenolic acid	1.45	6.84
Linezolid	1.59	7.53
Vancomycin	0.826	3.90
Caffeine	0.212	1.00

### Comparison of the RMS and conventional methods for determining the concentration of each drug in the control serum samples

**Fig. 4:**
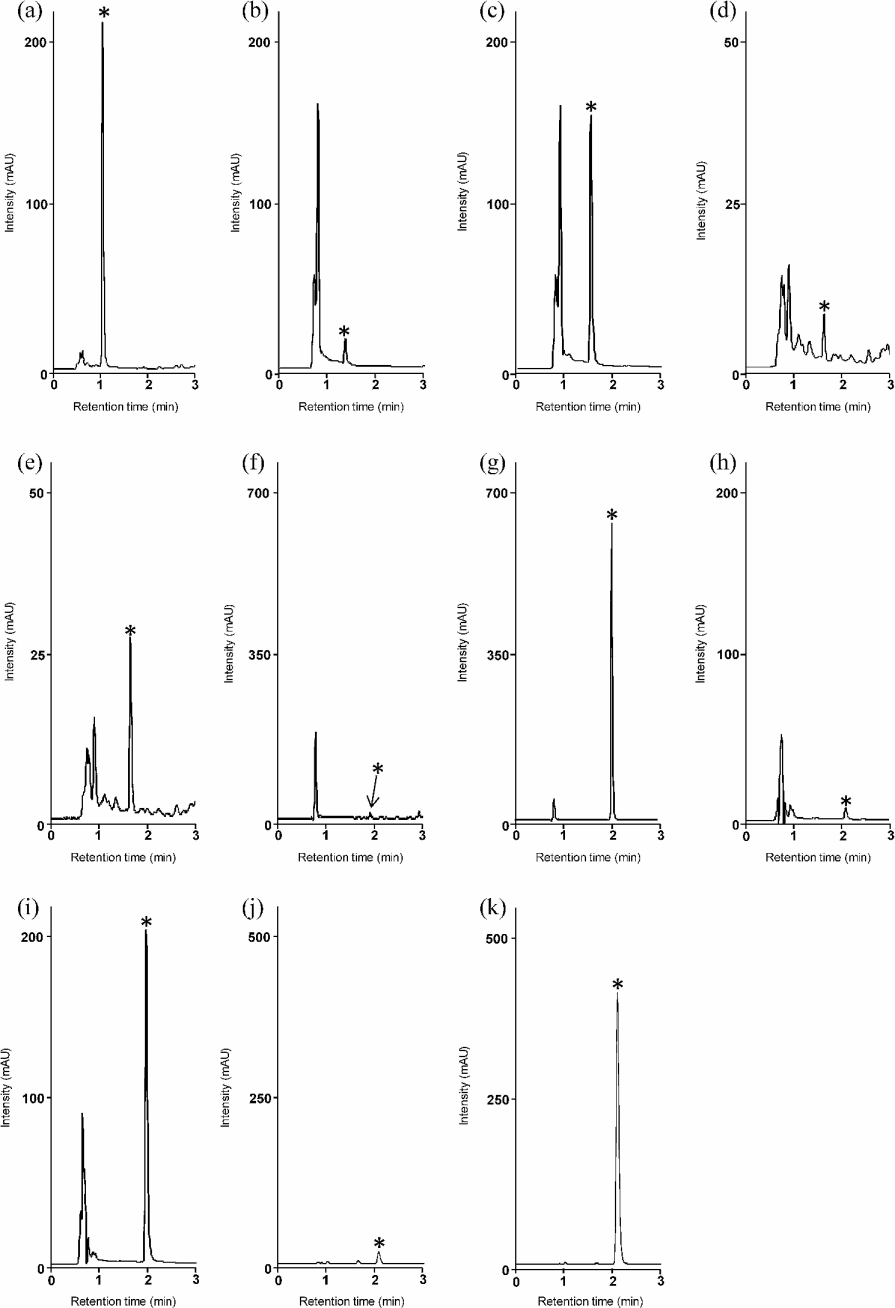
Representative HPLC chromatograms of control serum samples spiked with analyte (**a**) Sample 1, (**b**) Sample 2, (**c**) Sample 3, (**d**) Sample 4, (**e**) Sample 5, (**f**) Sample 6, (**g**) Sample 7, (**h**) Sample 8, (**i**) Sample 9, (**j**) Sample 10, (**k**) Sample 11 *: Peak of analyte for quantification

We validated the accuracy of the obtained RMSs by determining the concentration of each drug in samples spiked individually with one or two concentrations of carbamazepine, phenytoin, voriconazole, meropenem, mycophenolic acid, or linezolid by HPLC using RMS (the proposed method). The results were compared with those obtained by the absolute calibration method (the conventional method). The chromatograms of the drug-spiked control serum samples are shown in Table 3; Fig. 4 shows the concentration of each drug in the samples obtained by HPLC using RMS and the conventional method. Quantitative values obtained using the proposed and conventional methods with different non-analyte reference materials were almost the same for all samples spiked with each drug. In addition, no significant differences were observed, with a good RSD of less than 5.5%, indicating that the target compounds in each sample can be accurately quantified by HPLC using the non-analyte reference material and its corresponding RMS.

**Table 3:** Comparison of analyte content in samples, determined by two methods

	Sample name	Spiked concentration (μg/mL)	RMS method (Calibrant: Carbamazepine)		RMS method (Calibrant: Caffeine)		Conventional method	
			Concentration (μg/mL)	RSD (%)	Concentration (μg/mL)	RSD (%)	Concentration (μg/mL)	RSD (%)
Carbamazepine spiked	Sample 1	20	21.1	1.9	21.1	1.9	21.1	1.9
Phenytoin spiked	Sample 2	2	2.1	0.9	2.1	0.9	2.1	0.9
	Sample 3	20	18.0	2.5	18.1	2.5	18.1	2.5
Voriconazole spiked	Sample 4	1	1.1	1.0	1.0	1.1	1.1	1.0
	Sample 5	5	5.1	2.0	5.2	1.9	5.2	1.9
Meropenem spiked	Sample 6	0.5	0.4	1.4	0.4	1.4	0.4	1.4
	Sample 7	100	85.5	2.8	85.9	2.8	85.5	2.8
Mycophenolic acid spiked	Sample 8	1	1.1	0.5	1.1	0.5	1.1	0.5
	Sample 9	20	17.5	1.3	17.5	1.3	17.5	1.3
Linezolid spiked	Sample 10	2	2.3	0.7	2.3	0.7	2.3	0.7
	Sample 11	50	54.9	5.5	54.8	5.5	54.7	5.5

## Conclusions

As described in the literature [9, 32], carbamazepine, phenytoin, voriconazole, lamotrigine, mycophenolic acid, and vancomycin are in demand for measurement as TDM target drugs. Among them, voriconazole and lamotrigine are drugs for which no bioassay-based assay kits exist and must be measured by separation analysis methods. On the other hand, meropenem, linezolid, and caffeine are not TDM drugs; however, meropenem represents a broad-spectrum antimicrobial, and is needed to control its appropriate use [33]. In addition, caffeine should be measured quickly in emergencies to determine its intoxication [34]. Furthermore, linezolid has recently been reported to increase blood levels of linezolid in patients with renal impairment, increasing the risk of thrombocytopenia [35]. Based on this background, if the RMS method, which can measure multiple drugs from one accurate reference substance, can be applied to the measurement of these blood drug concentrations, it can be expected to improve the accuracy of blood concentration measurements at their own institutions, further improve treatment outcomes, and prevent adverse effects. Therefore, in this study, we established and validated an HPLC method using RMS for carbamazepine, phenytoin, voriconazole, lamotrigine, meropenem, mycophenolic acid, linezolid, vancomycin and caffeine to aid in the determination of blood drug concentrations to optimize drug dosage and administration. Our results demonstrate that the RMS method has good accuracy, precision, and linearity in the assessed concentration range. In addition, all quantitative values obtained using the RMS and conventional methods were almost the same in samples spiked with each drug. The RMS method thus appears to be useful for determining the concentrations of nine drugs for TDM and pharmaceutical quantification. Carbamazepine and caffeine, which are stable and for which certified reference materials with certified purities are readily available, were used as non-analyte reference materials in this RMS method. This method does not require an authentic reference material for the analyte to determine the concentration of each drug, reducing the need for and cost of the reference material for each analyte. ​​​​​​​In addition, the identification of each analyte can be achieved on the basis of the retention time because HPLC conditions are optimized for the analysis of each analyte. Moreover, meropenem and mycophenolic acid are unstable; therefore, this method reduces the risk of decomposition during storage and subsequent re-purchase. The proposed method holds promise for rapid and routine clinical analyses and as an alternative quantification method for the nine tested drugs.

## Data Availability

The datasets used and/or analysed during the current study are available from the corresponding author on reasonable request.
